# Abnormal activity of default mode network in GERD patients

**DOI:** 10.1186/1471-2202-14-69

**Published:** 2013-07-11

**Authors:** Huihui Sun, Ying Chen, Xiaohu Zhao, Xiangbin Wang, Yuanxi Jiang, Ping Wu, Yinhan Tang, Qingwei Meng, Shuchang Xu

**Affiliations:** 1 Department of Gastroenterology, Tongji Institute of Digestive Diseases, Tongji Hospital, Tongji University School of Medicine, No. 389 Xin Cun Road, Shanghai 200065, China; 2 Imaging Department, Tongji Hospital, Tongji University School of Medicine, Shanghai 200065, China; 3 Department of Clinical Nutrition, Tongji Hospital, Tongji University School of Medicine, Shanghai 200065, China

## Abstract

**Background:**

Abnormal processing of esophageal sensation at the level of the central nervous system has been proven to be involved in gastroesophageal reflux disease (GERD). However, most studies were focused on the possible functions of perceptual processing related network during task status, little attention has been paid to default mode network, which has been manifested to be important in the pathogenesis of many diseases. In our study, we compared the brain activity characteristic in GERD patients with the healthy subjects (HS) at baseline, looking for whether activities of default mode network were abnormal in GERD patients and attempting to identify their possible roles in GERD. In present study, fractional amplitude of low-frequency fluctuation was adopted to detect the brain activities at baseline. Group-level analyses were conducted by one-sample t test within groups (voxel thresholds were p < 0.001 and cluster level >42, corrected P < 0.05) and independent-samples t test between groups (p < 0.01 and cluster level >90, corrected P < 0.05) using SPM5.

**Results:**

The predominant activity area in both groups mainly located in default mode network such as medial superior frontal gyrus, precuneus, posterior cingulate gyrus, etc. However, the activities of precuneus and posterior cingulate gyrus were significantly lower in GERD patients than those in the HS.

**Conclusions:**

The activities of precuneus and posterior cingulate gyrus of default mode network in GERD patients were significantly lower compared to the HS, suggesting abnormal activities of brain regions in default mode network may be involved in pathophysiology of GERD symptom generation.

## Background

Gastroesophageal reflux disease (GERD) is defined as “symptoms or complications associated with regurgitation from the stomach and (or) the duodenum to the esophagus.” Its typical symptoms include heartburn and sour regurgitation. It has been estimated the prevalence of GERD symptoms in the general population ranges from 34% to 40% by using postal questionnaires [1,2]. However, the pathogenesis of GERD has not been fully elucidated. Several mechanisms have been proposed for its symptom generation, including acid reflux, motor dysfunction, and esophageal hypersensitivity etc. [3], in which visceral hypersensitivity received great attention in recent years especially in patients with non-erosive reflux disease.

GERD patients have esophageal hypersensitivity which has been reported in previous studies. For example, Fass et al. [4] performed a modified acid perfusion test in GERD patients and confirmed the presence of acid hypersensitivity. Miwa et al. [5] also reported that there was esophageal hypersensitivity in GERD patients in Japanese population. Hypersensitivity, defined by reduced pain and discomfort thresholds, was caused by abnormal mediation of sensation. In recent years, the nervous system termed as ‘brain–gut axis’, which mediates gastrointestinal sensation, has been attracting more attention. The brain–gut axis has mainly three levels: the enteric nervous system, the spinal cord and the brain. Up-regulation of sensation at any of these three levels could explain the hypersensitivity. During the past 10 years, by using brain imaging techniques, abnormal processing of visceral sensation at the level of central nervous system (CNS) in GERD has attached great concern. Currently, several different cerebral regions including the sensory/motor cortex, parieto-occipital region, prefrontal cortex (PFC), anterior cingulate cortex (ACC), insular cortex (IC) and cerebellum have been reported to be involved in the cerebral processing of visceral afferent signals, in which PFC, ACC and IC have been reported to be involved in esophageal hypersensitivity [6,7]. However, the majority of these neuroimaging studies were performed on the possible functions of brain networks involved in the perceptual process (mainly consisted of positively activated brain regions) in GERD, few studies have been focused on negatively activated brain regions. Recently, Van Oudenhove et al. [8] studied deactivated regional brain during gastric balloon distension, and found that bilateral occipital, lateral parietal and temporal cortex as well as medial parietal lobe (posterior cingulate cortex and precuneus) significantly deactivated in Baseline–maximal distension (C1–C4) and correlated negatively with intra-gastric pressure and epigastric sensation score. Interestingly, above regions have previously been proven to be part of the default mode network (DMN), in which the precuneus/ posterior cingulate cortex (PCC) plays a pivotal role in it [9]. DMN is currently defined as one network which is consisted of regions routinely exhibiting task-related deactivations. Its functions include central nervous system monitoring for internal and external environment, emotional processing, self-introspection, maintaining awareness of consciousness, reviewing episodic memory and so on [10], abnormal activity of which has been shown to involve in many diseases such as Alzheimer’s disease, schizophrenia [11,12]. Nevertheless, the role of DMN in gastrointestinal diseases has traditionally received little attention. Furthermore, most of these studies focused on the CNS responses to visceral stimulations, while few attentions were paid to brain activity at baseline. In fact, the resting energy consumption outdistances task or stimulus-related increases in neural metabolism. Spontaneous neural activity at baseline can provide more comprehensive information on how the brain behaves.

In our study, we analyzed the resting data from our previously published functional magnetic resonance imaging (fMRI) study in GERD patients and the HS [13], by adopting fractional amplitude of low-frequency fluctuation (fALFF). fALFF is one of the measures of spontaneous low-frequency oscillations (LFO), which can be detected with resting-state fMRI. Through this approach, we observed the activity change pattern of DMN in GERD, and attempt to identify the potential function of DMN in GERD.

## Results

### Baseline characteristics of the subjects in each group

Total forty-two subjects met the inclusion criteria and entered the study, including twenty-nine GERD patients (12 women, mean age 46.45) and thirteen HS (5 women, mean age 38.46). Detailed demographical features of all patients and HS were shown in Table 1. The age of GERD patients was significantly higher compared with HS. No differences among these groups were found with regard to sex composition.

**Table 1:** Demographical parameters of overall GERD patients and HS

**Demographics parameters**	**GERD**	**HS**	**P**
Patients, n	29	13	
Female patients, n (%)	12(41.4%)	5(38.46)	0.859
Mean Age (±SD), years	46.45(±25.07)	24.33(±2.93)	0.004

Note: Data are presented as mean and SD (SD, standard deviation), P ≤0.05 represents significantly different.

### Significant brain activities at baseline in the HS

**Figure 1:**
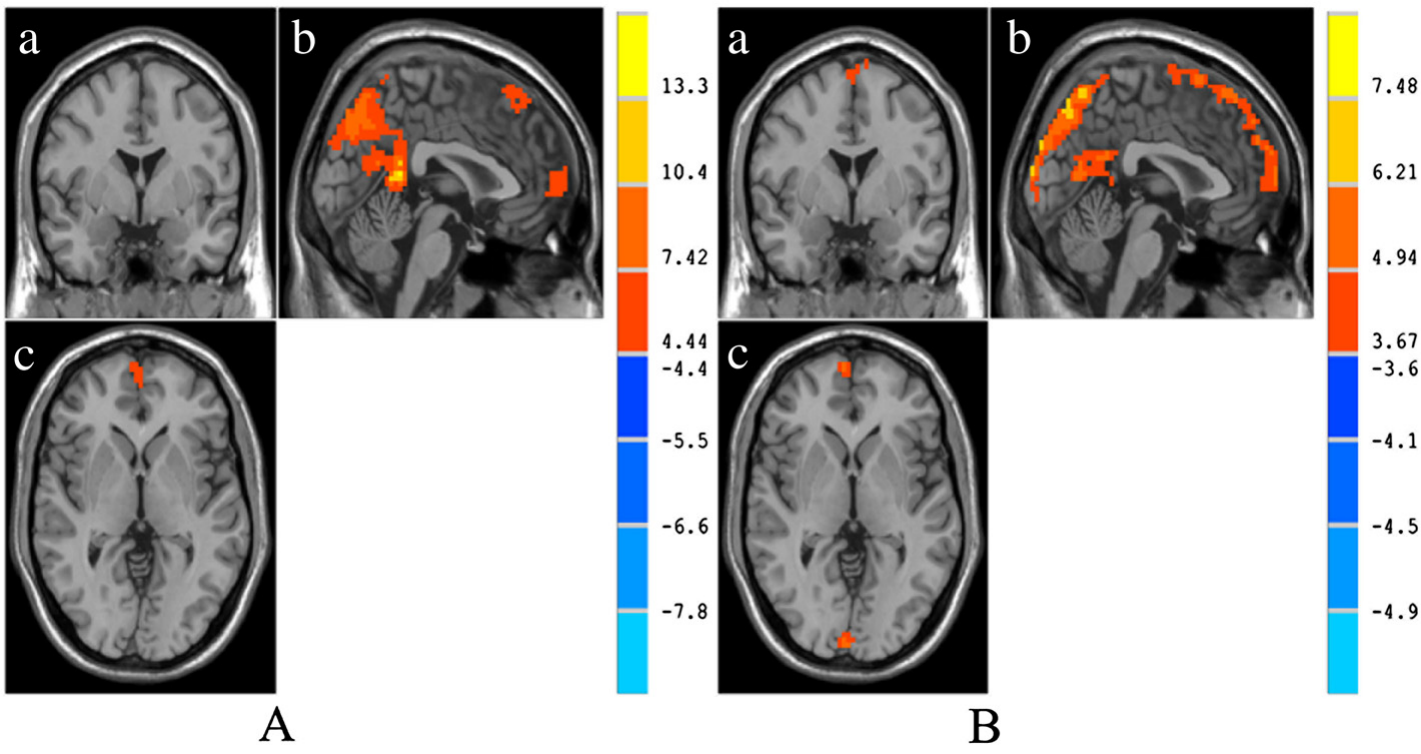
**Brain activities in HS (A) and GERD (B) by fALFF. (a)** anteroposterior image, **(b)** coronal image, **(c)** transverse image. The cold color represents the fALFF of brain regions which are lower than the mean of the whole brain, and the warm color represents the fALFF of brain regions which are higher than the mean of the whole brain.

The significant activity regions of both groups at baseline were shown in Figure 1. Within experimental groups a single voxel threshold was set at p < 0.001 and cluster level >42. In HS, fALFF was higher and dominant in precuneus, PCC, left middle frontal gyrus, and bilateral medial superior frontal gyrus (the detail Montreal Neurological Institute (MNI) peak coordinates, volume, peak activity intensity of each brain region were showed in Table 2).

**Table 2:** Significant brain activities in HS at baseline

**Brain region**	**MNI peak coordinates (X,Y,Z)**			**Volume**	**Peak activity intensity**
Right medial superior frontal gyrus	9	6-	3	29	7.253
Precuneus	3	−69	42	256	16.8031
Posterior cingulate gyrus	3	69	42	195	16.8031
Left middle frontal gyrus	−27	33	51	35	11.0488
Left medial superior frontal gyrus	−3	27	60	28	7.2166

Note: Statistical threshold was set as t = 4.437, p < 0.001 and cluster level >42 voxels.

*MNI* Montreal Neurological Institute, *GERD* Gastroesophageal Reflux Disease.

### Significant brain activities at baseline in GERD patients

In GERD patients, fALFF was higher and dominant in precuneus, PCC, left middle frontal gyrus, bilateral medial superior frontal gyrus, and left supplementary motor area(SMA) (the detail material of each brain region could be found in Table 3).

**Table 3:** Significant brain activities in GERD at baseline

**Brain region intensity**	**MNI peak coordinates (X, Y, Z)**			**Volume**	**Peak activity**
Precuneus	3	−69	57	120	9.2972
Posterior cingulate gyrus	0	−66	12	63	6.3883
Right medial superior frontal gyrus	3	63	9	67	6.6306
Left middle frontal gyrus	−12	33	51	30	6.0696
Left medial superior frontal gyrus	−3	30	57	74	6.6797
Left supplementary motor area	−3	15	69	52	5.598

Note: Statistical threshold was set as t = 3.6739, p < 0.001 and cluster level >42 voxels.

*MNI* Montreal Neurological Institute, *GERD* Gastroesophageal Reflux Disease.

### Comparison of brain activities at baseline between HS and GERD patients

**Figure 2:**
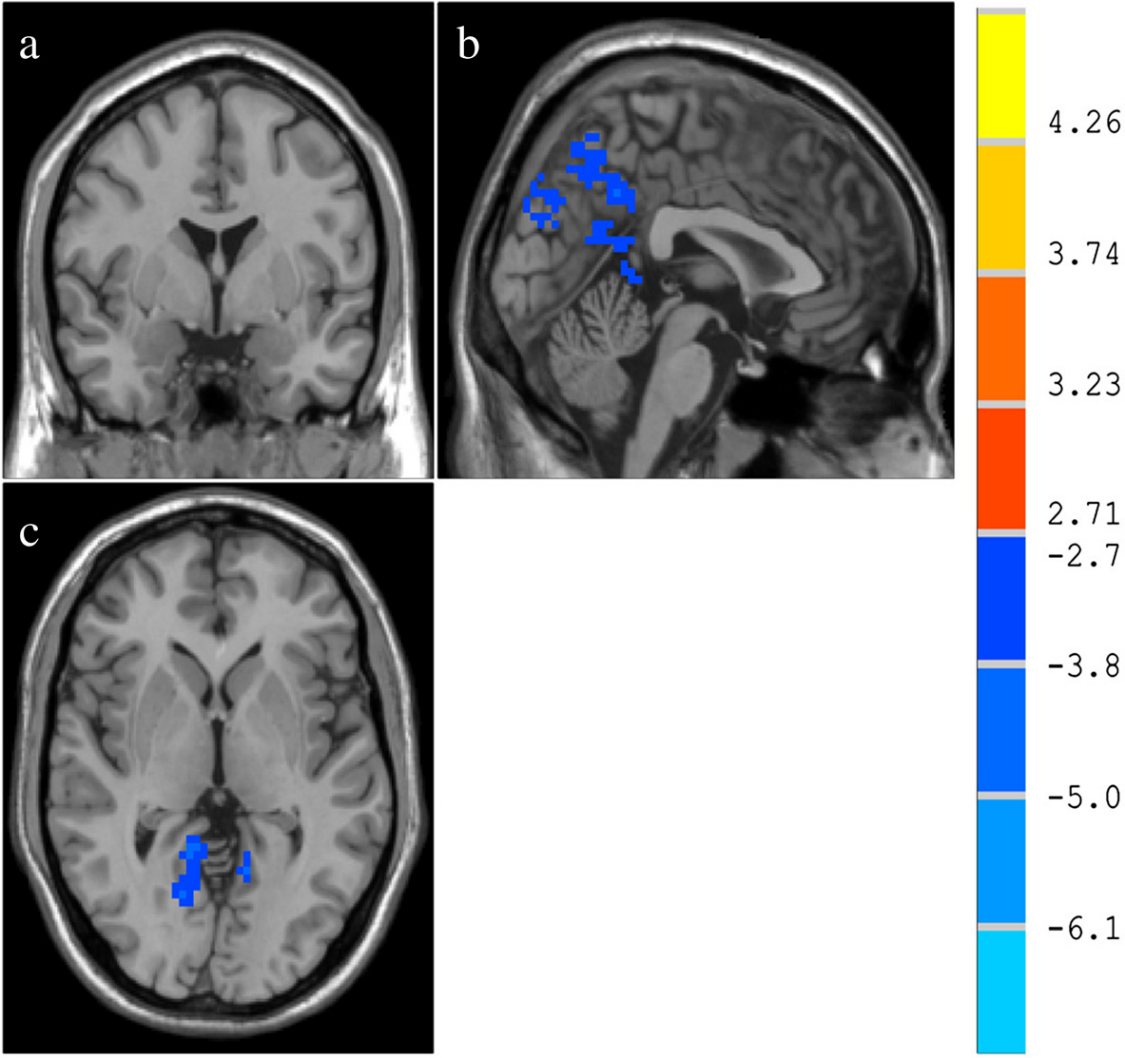
**Comparison of brain activities between the HS and GERD patients. (a)** anteroposterior image, **(b)** coronal image, **(c)** transverse image. The cold color represents the fALFF of brain regions which are lower in GERD than HS. The fALFF of GERD is significantly lower in the bilateral precuneus and PCC compared with HS (p < 0.01 and cluster level >90 voxels).

The voxel threshold at P < 0.01 and a minimum cluster size of 90 voxels between both groups were considered significantly different. Regions that showed significant differences in fALFF between GERD patients and the HS were shown in Table 4. Compared with HS, the fALFF of precuneus and PCC was significantly lower in GERD patients (Figure 2).

**Table 4:** Comparison of brain activities between HS and GERD at baseline

**Brain region**	**MNI peak coordinates (X,Y,Z)**			**Volume**	**Peak activity intensity**
Precuneus	−9-	54	9	256	−7.321
Posterior cingulate gyrus	−9-	54	9	251	−7.321

Note: Statistical threshold was set as t = 2.7116, p < 0.01 and cluster level >90 voxels.

*MNI* Montreal Neurological Institute, *GERD* Gastroesophageal Reflux Disease.

## Discussion

This study reveals the significant difference of cerebral activities at baseline between GERD patients and HS for the first time. The different activities mainly located at brain areas relevant to the DMN.

In our study, the significant activity regions of the HS at baseline mainly located in DMN. The term “default mode of brain function” was coined by Raichle et al. [14] to describe the baseline state in the human brain. Subsequently, Fox et al. [10] redefined DMN as task-negative network, the activity of which was greater at rest than that under various goal-directed tasks.

In consideration of the following reasons: (1) by functional imaging investigations, brain regions (especially precuneus/PCC) in DMN showed an elevated level of metabolic activity at baseline [14]; (2) activity of DMN in many stimulus tasks decreased compared with resting state, DMN has received much investigative attention by functional imaging studies as an interesting brain network in several diseases [15,16]. In previous studies, DMN has been manifested to be involved in the central nervous system monitoring for internal and external environment, emotional processing, self-introspection, maintaining awareness of consciousness, reviewing episodic memory and so on.

In GERD patients, the significant activity areas were also mainly located at DMN. GERD is a common gastrointestinal disorder. As we all know, many factors can contribute to the occurrence of GERD, but the pathogenesis of which remains to be elucidated. Previous studies have shown that visceral hypersensitivity was associated with GERD, in which abnormal processing of visceral sensation at the level of the CNS received great attention. For example, Kern et al. [17] performed a esophageal acid exposure test in GERD patients and found the cortical activity associated with liminal/perceived esophageal acid exposure in GERD patients occurred more rapidly and with greater intensity than the activity measured in the HS. Wang et al. [3] also confirmed that GERD patients and HS showed distinctly different activation patterns (mainly in IC, ACC, PFC), and speculated GERD patients have different patterns of visceral perception in esophageal acid stimuli test. In our previous study, we likewise found that the activated intensity of the ACC, the right side of the dorsolateral prefrontal cortex and the left side of the IC in GERD patients were significantly different from the HS [13]. Therefore, visceral hypersensitivity caused by central sensitivity was supposed to be one important mechanism in symptom generation of GERD, in which IC, ACC have consistently been shown to be abnormally activated in GERD during all kinds of stimulus tasks [6]. However, the fALFF of ACC and IC were not significantly increased at baseline in GERD. This phenomenon may suggest that above regions are not abnormal at rest, just more sensitive to stimulus during task state.

In comparison between two groups, we found the activity of GERD at baseline was remarkably lower in precuneus and PCC of DMN. In recent years, the precuneus and PCC are as key components of DMN, receiving much investigative attention by functional imaging studies [18]. However, the majority of studies have focused on their roles in fundamental cognitive function, and relatively little attention was paid to their roles in sensory regulation. By using fMRI, Lawal et al. [19] compared the activation topography of the cingulate cortex in response to modified anal sphincter contraction in males and females, and discussed the involvement of the PCC in visceral sensation. Their findings were in concordance with previous reports [20], suggesting the PCC was involved in the modulation of visceral sensation. Precuneus has also been considered to be possibly involved in visceral sensation in several visceral stimulus studies [21,22]. Recently, in the study relevant to deactivated brain regions during gastric balloon distension, Van Oudenhove et al. [8] found that bilateral occipital, lateral parietal and temporal cortex, PCC and precuneus which were relevant to the DMN significantly deactivated in baseline–maximal distension,and negatively correlated with Intra-gastric pressure and epi-gastric sensation score. Their finding suggests that the decreased activity of DMN may involve in pathological generation of visceral hypersensitivity. In our study, the baseline activities of precuneus and PCC in DMN were also found to be significantly lower in GERD patients compared with HS, which means that the abnormal activity of DMN at baseline may involve in the pathophysiology of GERD, by modulating visceral sensation. Interestingly, except precuneus and PCC, the activities of other brain regions of DNM in GERD patients were not significantly lower compared to HS. The possible reasons might lie in: 1) precuneus and PCC are amongst the brain structures displaying the highest resting metabolic rates (hot spots) [18], so their reactions to diseases are more sensitive than other brain regions of DMN; 2) maybe only precuneus and PCC, but no other regions in DMN were involved in GERD.

Besides DMN, the mean fALFF of SMA was also found to be significantly higher than the mean of whole brain in GERD patients. SMA has been confirmed to be involved in central processing of movement intention, motor preparation and visceral pain [23,24]. Furthermore, SMA was found to be significantly activated in patients with hypersensitivity in many visceral task stimulus studies [25,26], suggesting that abnormal activity of SMA may play an important role in the pathophysiology of GERD.

In our study, the age of GERD patients was significantly higher than HS. As known, age has non-negligible effect on brain activity. Previous studies have shown that with age increasing, the activity of DMN changes, but it only happens when there is a big age span [27]. In order to eliminate the effect of age on the comparison of brain activities between the two groups, age was regressed as a covariate in our independent-samples t test. The results showed that the significantly different areas between two groups were similar before and after regression of age factor, which mainly located at precuneus and PCC. In our experiment, the objects enrolled were mostly between 20 and 50 years old. In this age period, the DMN is relatively steady and the effect of age can be ignored [28]. Therefore, the differences of DMN activity between GERD patients and HS were mainly caused by disease, not the age factor.

## Conclusions

The activities of precuneus and PCC in DMN were significantly lower in GERD compared to HS. We infer that the decreased activity of DMN at baseline may be involved in the pathological generation and aggravation of GERD symptoms, through modulating visceral sensation. Whether precuneus and PCC are only abnormal areas or just more sensitive than other regions of DMN in GERD remains to be elucidated. In addition, the reason why the activity of DMN is abnormal in GERD patients at baseline also needs further investigations.

## Methods

### Subjects

Thirty-nine patients with typical reflux symptoms who visited Shanghai Tongji Hospital between April 2007 and January 2008 were enrolled in the study. The patients were diagnosed and identified based on a reflux questionnaire, endoscopy, proton pump inhibitor (PPI) test, and 24 h esophageal pH monitoring. The average age of the GERD group (twenty two males and seventeen females) was 48.23 ± 11.26 years old. The inclusion criteria was as follows: (1) between 18 and 65 years of age, regardless of gender; (2) with typical reflux symptoms lasting for at least 3 months or extra-esophageal manifestation that emerged simultaneously; (3) diagnosed as reflux esophagitis by endoscopy; or a negative endoscopic examination, but Reflux Disease Questionnaire (RDQ) score higher than 12; or a negative endoscopic examination, but a positive 24 h esophageal Ph monitoring test;or a negative 24 h pH monitoring test, but the positive PPI test. The exclusion criteria were as follows: (1) a peptic ulcer (regardless of the period) or erosive duodenitis by endoscopy; (2) a tumor in the digestive tract observed by endoscopy; (3) a history of operation on the digestive tract; and (4) a suspected or confirmed malignant tumor. Fifteen healthy volunteers (seven males and eight females) were enrolled in this study. All of the healthy volunteers were undergraduates or graduates and the average age of the healthy volunteers (seven males and eight females) was 23.67 ± 1.80 years. No accumulated systemic disease in the esophagus and no abnormalities were observed in any of the volunteers by endoscopy. All the subjects were right-handed. The subjects had not taken any medication that would influence visceral sensation within 1 week prior to participation in the study, nor had the subjects recently taken any medication affecting the nervous system. No metal was present in the bodies of the subjects and no encephaloncus tumor or other diseases in encephalo. The subjects had no medical records of neural or mental disease and no medical record of a head injury. The Tongji Hospital Ethics Committee approved all studies. All subjects signed an informed consent form before the study started.

### Study protocol

In this study, we analyzed the data that has been used in previous studies [13]. The protocol has been previously described in detail [13]. Briefly, in this study, we used an improved block-design model in which there was a long time task chunk and a resting chunk. The experiment was composed of a resting chunk (A, 8 min), normal saline perfusion (B, 8 min), acid perfusion (C, 8 min) and then normal saline perfusion again (D, 8 min) for a cumulative time of 32 min. Each chunk was divided into 160 scans and every scan contained 3 s for a total of 640 scans. In present study, we only analyzed the resting data.

### Data acquisition

All the fMRI scans were performed in the fMRI department of Tongji hospital. All fMRI data were obtained on a 1.5-T superconductive scanner (Marconi EDGE ECLIPSE, USA). During the scanning session, the subjects were told to stay relaxed, close their eyes and not to move their head. Rubber earplugs were used to lower the noise and foam pads were placed to head to reduce the head movements. The experiment began after each subject adapted to the environment of the scanning room.

BOLD-fMRI was collected axially using an echo-planar imaging (EPI) sequence with the following parameters: thickness/gap (THK/GAP) = 6/1 mm, voxel = 3.75 mm × 3.75 mm. Repetition time (TR)/echo time (TE)/flip angle (FA) = 1930 ms/40 ms/90°, field of view (FOV) = 24 cm × 24 cm, matrix =64 × 64, NEX = 1.

### Data preprocessing

SPM5(statistical parametric mapping 5) software (http://www.fil.ion.ucl.ac.uk/spm), REST(resting state fMRI data analysis toolkit) software (http://www.restfmri.net/forum/REST_V1.8), and Dparsf (data processing assistant for resting-state fMRI) software (http://www.restfmri.net/forum/DPARSF) were used for preprocessing and statistical analysis. The first 10 images were discarded in consideration of magnetization equilibrium. The remaining 150 images were corrected for the acquisition time delay among different slices, and then the images were realigned to the first volume for head-motion correction. The fMRI images were further spatially normalized to the MNI EPI template. Spatially smooth was performed using a Gauss kernel of 6 mm to reduce space noise.

The time course of head motion was obtained by estimating the translations in each direction and the rotations in angular motion about each axis for each of the 150 consecutive volumes. Ten GERD patients and two healthy volunteers were excluded for a maximum displacement of more than 3 mm (larger than the size of a voxel in plane) at each axis and an angular motion of more than 3° for each axis.

### Data processing

After preprocessed in SPM5, the linear trend was removed by Dparsf. The analysis procedure for fALFF was carried out according to the method of Zou et al. [29]. For the time series of each voxel, the sum of amplitudes within a low frequency range (0.01–0.08Hz) was calculated. The fALFF was then computed as the fractional sum of amplitudes within the low frequency range that was divided by the sum of amplitude across the entire frequency range (0–0.25Hz). The subject-level voxel-wise fALFF maps were standardized into subject-level by dividing the mean voxel-wise fALFF obtained for the entire brain. Mean fALFF maps was obtained and used for statistical analysis.

### Statistical analysis

Group-level analyses of the fALFF maps were conducted by one-sample t test (detect the activity in GERD and normal controls, respectively) and independent- samples t test (detect the difference between two groups) using REST. In order to eliminate the effect of age on the comparison of brain activities between the two groups, age was regressed as a covariate in independent-samples t test. The results were corrected using the AlphaSim program in REST software which is based on the Monte Carlo simulation in AFNI [30]. The full width at half maximum (FWHM) was estimated by using the gray matter mask. The estimated FWHM were about 11 mm in one-sample t tests and 9 mm in independent-samples t tests. 1000 iterations were performed. Cluster connection radius was set as 5 mm. For a corrected significant level of p < 0.05, the voxelwise intensity threshold was set at p < 0.001 and the cluster size threshold at 42 voxels in one-sample t test and the voxelwise intensity threshold was set at p < 0.01 and the cluster size threshold at 90 voxels in independent-samples t test.

## Abbreviations

GERD: Gastroesophageal reflux disease; HS: Healthy subjects; CNS: Central nervous system; PFC: Prefrontal cortex; ACC: Anterior cingulate cortex; IC: Insular cortex; DMN: Default mode network; PCC: Posterior cingulate cortex; fMRI: Functional magnetic resonance imaging; fALFF: Fractional amplitude of low-frequency fluctuation; LFO: Spontaneous low-frequency oscillations; SMA: Supplementary motor area; MNI: Montreal Neurological Institute; FWHM: Full width at half maximum.

## Competing interests

This study was supported in part by grants 10411968100 from Science and Technology Commission of Shanghai Municipality. The related authorities and the authors declare that they all have no competing interest.

## Authors’ contributions

HHS performed the research, analyzed the data, and wrote this manuscript. YC deigned the study, performed the research. XHZ designed the study, revised the paper critically. XBW analyzed the data. YXJ performed the research. PW revised the paper. QWM and YHT edited the manuscript. SCX designed the study, performed much of the research, and revised the paper critically. All authors read and approved the final manuscript.

## References

[B1] Corder A Jones R Sadler G Daniels P Johnson C Heartburn, oesophagitis and Barrett’s oesophagus in self-medicating patients in general practice Brit J Clin Pract 1996 50 245 248 8794600

[B2] Ronkainen J Aro P Storskrubb T Johansson SE Lind T Bolling-Sternevald E Graffner H Vieth M Stolte M Engstrand L High prevalence of gastroesophageal reflux symptoms and esophagitis with or without symptoms in the general adult Swedish population: a Kalixanda study report 2005 40 275 285 10.1186/1471-230X-11-28 15932168

[B3] Wang K Duan LP Zeng XZ Liu JY Xu-Chu W Differences in cerebral response to esophageal acid stimuli and psychological anticipation in GERD subtypes-An fMRI study BMC Gastroentero 2011 11 28 [Epub ahead of print] 10.1016/S0016-5085(98)70014-9 PMC3073936 21439078

[B4] Fass R Naliboff B Higa L Johnson C Kodner A Munakata J Ngo J Mayer EA Differential effect of long-term esophageal acid exposure on mechanosensitivity and chemosensitivity in humans Gastroentero 1998 115 1363 1373 10.1111/j.1365-2036.2004.01990.x 9834263

[B5] Miwa H Minoo T Hojo M Yaginuma R Nagahara A Kawabe M Ohkawa A Asaoka D Kurosawa A Ohkusa T Oesophageal hypersensitivity in Japanese patients with non-erosive gastro-oesophageal reflux diseases Aliment Pharm Therap 2004 20 112 117 10.1111/j.1365-2036.2004.01990.x 15298616

[B6] Van Oudenhove L Coen SJ Aziz Q Functional brain imaging of gastrointestinal sensation in health and disease World J Gastroentero 2007 13 3438 3445 10.1053/j.gastro.2007.10.008 PMC4146779 17659690

[B7] Kern M Antonik SJ Mepani R Lawal A Shaker R 907 GERD alters the functional brain connectivity models associated with acid stimulation Gastroentero 2008 134 131 10.1111/j.1365-2982.2008.01196.x

[B8] Van Oudenhove L Vandenberghe J Dupont P Geeraerts B Vos R Bormans G Van Laere K Fischler B Demyttenaere K Janssens J Cortical deactivations during gastric fundus distension in health: visceral pain-specific response or attenuation of ‘default mode’brain function? A H215O‒PET study Neurogastroent Motil 2009 21 259 271 10.1016/j.neuroimage.2008.05.059 19019011

[B9] Fransson P Marrelec G The precuneus/posterior cingulate cortex plays a pivotal role in the default mode network: Evidence from a partial correlation network analysis Neuroimage 2008 42 1178 1184 10.1073/pnas.0504136102 18598773

[B10] Fox MD Snyder AZ Vincent JL Corbetta M Van Essen DC Raichle ME The human brain is intrinsically organized into dynamic, anticorrelated functional networks P Natl Acad Sci USA 2005 102 9673 9678 10.1073/pnas.2235925100 PMC1157105 15976020

[B11] Lustig C Snyder AZ Bhakta M O’Brien KC McAvoy M Raichle ME Morris JC Buckner RL Functional deactivations: change with age and dementia of the Alzheimer type P Natl Acad Sci USA 2003 100 14504 14509 10.1176/appi.ajp.164.3.450 PMC283621 14608034

[B12] Garrity A Pearlson G McKiernan K Lloyd D Kiehl K Calhoun V Aberrant “default mode” functional connectivity in schizophrenia Am J Psychiat 2007 164 450 457 10.1016/j.ejrad.2010.03.025 17329470

[B13] Xu SC Zheng FF Zhao XH Chen Y Kong X Wang C Zhu LR Wang ZY Brain processing of visceral sensation upon esophageal chemical stimulation in different types of GERD Eur J Radiol 2010 75 352 359 10.1073/pnas.98.2.676 20434286

[B14] Raichle ME MacLeod AM Snyder AZ Powers WJ Gusnard DA Shulman GL A default mode of brain function P Natl Acad Sci 2001 98 676 10.1038/35094500 PMC14647 11209064

[B15] Gusnard DA Raichle ME Raichle M Searching for a baseline: functional imaging and the resting human brain Nat Rev Neurosci 2001 2 685 694 10.1002/hbm.21034 11584306

[B16] Luo C Li Q Lai Y Xia Y Qin Y Liao W Li S Zhou D Yao D Gong Q Altered functional connectivity in default mode network in absence epilepsy: a resting‒state fMRI study Hum Brain Mapp 2011 32 438 449 10.1016/S0016-5085(98)70013-7 21319269 PMC6870112

[B17] Kern MK Birn RM Jaradeh S Jesmanowicz A Cox RW Hyde JS Shaker R Identification and characterization of cerebral cortical response to esophageal mucosal acid exposure and distention Gastroentero 1998 115 1353 1362 10.1093/brain/awl004 9834262

[B18] Cavanna AE Trimble MR The precuneus: a review of its functional anatomy and behavioural correlates Brain 2006 129 564 583 10.1152/ajplung.00217.2005 16399806

[B19] Lawal A Kern M Sanjeevi A Hofmann C Shaker R Cingulate cortex: a closer look at its gut-related functional topography Am J Physiol - Gastr L 2005 289 722 730 10.1016/0361-9230(82)90006-5 16160081

[B20] Henke PG Savoie RJ The cingulate cortex and gastric pathology Brain Res Bull 1982 8 489 492 10.1053/j.gastro.2006.05.014 7116197

[B21] Naliboff BD Berman S Suyenobu B Labus JS Chang L Stains J Mandelkern MA Mayer EA Longitudinal change in perceptual and brain activation response to visceral stimuli in irritable bowel syndrome patients Gastroentero 2006 131 352 365 10.1016/j.pain.2007.01.032 16890589

[B22] Kano M Hamaguchi T Itoh M Yanai K Fukudo S Correlation between alexithymia and hypersensitivity to visceral stimulation in human Pain 2007 132 252 263 10.1053/j.gastro.2011.06.008 17360119

[B23] Coen SJ Kano M Farmer AD Kumari V Giampietro V Brammer M Williams SCR Aziz Q Neuroticism influences brain activity during the experience of visceral pain Gastroentero 2011 141 909 917 10.1053/j.gastro.2005.02.068 21699797

[B24] Yágüez L Coen S Gregory LJ Amaro E Altman C Brammer MJ Bullmore ET Williams SCR Aziz Q Brain response to visceral aversive conditioning: a functional magnetic resonance imaging study Gastroentero 2005 128 1819 1829 10.1053/j.gastro.2005.02.068 15940617

[B25] Aziz Q Thompson D Ng V Hamdy S Sarkar S Brammer M Bullmore E Hobson A Tracey I Gregory L Cortical processing of human somatic and visceral sensation J Neurosci 2000 20 2657 2663 10.1016/S0016-5085(97)70079-9 10729346 PMC6772246

[B26] Aziz Q Andersson J Valind S Sundin A Hamdy S Jones A Foster E Langstrom B Thompson D Identification of human brain loci processing esophageal sensation using positron emission tomography Gastroentero 1997 113 50 59 10.1016/j.neurobiolaging.2008.05.022 9207261

[B27] Sambataro FS Murty VP Callicott JH Tan HY Das S Weinberger DR Mattay VS Age-related alterations in default mode network: impact on working memory performance Neurobiol Aging 2010 31 839 852 10.1073/pnas.0135058100 18674847 PMC2842461

[B28] Greicius MD Krasnow B Reiss AL Menon V Functional connectivity in the resting brain: A network analysis of the default mode hypothesis P Natl Acad Sci USA 2003 100 253 258 10.1016/j.jneumeth.2008.04.012 PMC140943 12506194

[B29] Zou QH Zhu CZ Yang Y Zuo XN Long XY Cao QJ Wang YF Zang YF An improved approach to detection of amplitude of low-frequency fluctuation (ALFF) for resting-state fMRI: fractional ALFF J Neurosci Meth 2008 172 137 141 10.1371/journal.pone.0025031 PMC3902859 18501969

[B30] Song XW Dong ZY Long XY Li SF Zuo XN Zhu CZ He Y Yan CG Zang YF Rest: a toolkit for resting-state functional magnetic resonance imaging data processing PLoS One 2011 6 e25031 21949842 10.1371/journal.pone.0025031 PMC3176805

